# Biochemical Study of Bilberry Extract Potential in Preventing Retinal Damage in Rat Model of Diabetes Induced by Streptozotocin/Nicotinamide

**DOI:** 10.3390/life15071006

**Published:** 2025-06-25

**Authors:** Maja Petrović, Marija Trenkić, Marija Veselinović, Aleksandra Smiljković, Dušan Sokolović

**Affiliations:** 1 Ophthalmology Clinics, University Clinical Centre Niš, 18000 Niš, Serbia; drpetrovicmaja@gmail.com (M.P.); marija.trenkic@gmail.com (M.T.); drmarija.veselinovic@gmail.com (M.V.); todorovicaleksandra1991@gmail.com (A.S.); 2 Faculty of Medicine, University of Niš, 18000 Niš, Serbia; 3 Department of Biochemistry, Faculty of Medicine, University of Niš, 18000 Niš, Serbia

**Keywords:** bilberry extract, diabetes, retinal damage, VGEF

## Abstract

Type 2 diabetes mellitus is a growing global health concern, with diabetic retinopathy (DR) representing a major microvascular complication that contributes significantly to vision impairment. Oxidative stress plays a critical role in the pathogenesis of DR, which is associated with changes in vascularization-associated molecules, such as iNOS, VEGF, and MMP-9. The present study investigates the therapeutic potential of bilberry (*Vaccinium myrtillus* L.) extract—rich in anthocyanins—applied for 14 days on blood glucose levels, lipid profile, and retinal oxidative stress (lipid peroxidation (TBARS) and advanced oxidized protein products (AOPPs)) in a streptozotocin/nicotinamide (STZ/NA)-induced diabetes rat model. Results showed a significant reduction in non-fasting blood glucose, retinal TBARS, and AOPP levels, and normalization of VEGF and MMP-9 expression in bilberry-treated diabetic rats. Bilberry extract also partially improved lipid profile by lowering LDL levels. However, no significant effects on fasting glucose or serum insulin were observed. These findings suggest that bilberry extract may offer protective effects against oxidative retinal damage and could serve as a complementary approach in managing early diabetic retinopathy.

## 1. Introduction

Type 2 diabetes mellitus is a global burden with constantly increasing prevalence. It was estimated that there will be a 69% increase in the numbers of adults with diabetes in developing countries and a 20% increase in developed countries between 2010 and 2030 [1]. Diabetic retinopathy is a common microvascular complication of diabetes and the leading cause of blindness in the working-age population. It is identified in one-third of people with diabetes and associated with an increased risk of life-threatening vascular complications, including stroke and coronary heart disease [2]. Chronic hyperglycemia, as well as hypertension, and hyperlipidemia are known to contribute to the pathogenesis of diabetic retinopathy [3]. Several biochemical pathways have been proposed as a potential link between hyperglycemia and diabetic retinopathy, where the oxidative stress is believed to be the key mechanism in the initiation and progression of diabetic retinopathy [4]. In an animal model of diabetes, an increase in retinal thiobarbituric acid reactive substances (TBARSs) and inducible nitric oxide synthase (iNOS) levels has been reported [5]. An increase in TBARS is indicative of structural cell lipid damage, which points to a certain degree of retinal degermation. On the other hand, increased iNOS activity suggests a source for nitric oxide (NO) production, which is involved in numerous pathophysiological processes. Reactive oxygen species (ROS) cause damage to cellular structures by oxidizing lipids, proteins, and nucleic acids and are linked to the change in retinal levels of enzymes and growth factors. One of such molecules is vascular endothelial growth factor (VEGF), which causes an increase in vascular permeability in the ischemic retina and breakdown of the blood–retinal barrier, and it stimulates endothelial cell growth and neovascularization [4]. Also, matrix metalloproteinases (MMPs), including MMP-9, are enzymes involved in the degradation of extracellular matrix proteins and probably in the disruption of the blood–retinal barrier in diabetic retinopathy [6].

Bilberry or European blueberry (*Vaccinium myrtillus* L.) is one of the richest natural sources of anthocyanins which are believed to be responsible for the many health benefits of bilberry. Anthocyanins, water-soluble polyphenolic compounds, give bilberry its blue/black color and are responsible for its high antioxidant potential [7]. Although bilberry is promoted most commonly for improving vision and ocular disorders [8,9] it has been reported to lower blood glucose [10] and oxidative stress [11]. In the animal model of diabetes induced by alloxan bilberry extract (2 g/kg)-reduced blood glucose, elevated insulin levels and normalized serum lipid profile were observed [10]. Bilberry, one of the major sources of dietary anthocyanin intake, has been used in traditional European medicine for the treatment of disorders of the gastrointestinal tract and diabetes, which is proven in some clinical studies [12]. Thus, vaccinium fruits and extracts obtained from the fruits are promising for targeting different pathways associated with diabetes, and this has been proven both in preclinical and clinical settings [12].

The aim of the current study was to determine the effect of bilberry extract application to rats with streptozotocin/nicotinamide-induced diabetes mellitus on changes in blood glucose and lipid profile, as well as the changes in retinal-tissue oxidative stress, and levels of iNOS, VEGF, and MMP-9.

## 2. Materials and Methods

### 2.1. Chemicals

Methanol, formic acid, and acetonitrile were from Merck (Darmstadt, Germany), while the standard cyanidin 3-glucoside was from Polyphenols Laboratories AS (Sandnes, Norway). Aqueous solutions were prepared using an ultra-pure water, Milli-Q water (Millipore, Bedford, MA). Streptozotocin (STZ) and nicotinamide (NA) were purchased from Sigma Aldrich (St Louis, MO, USA). All other chemicals used for the experiment and biochemical analysis were of analytical grade and obtained from either Merck (Darmstadt, Germany) or Sigma Aldrich (St Louis, MO, USA). All solutions used in the research were prepared fresh daily before experimental procedures.

### 2.2. Bilberry Extract Preparation and Content Determination

Bilberry extract, prepared from fully ripe bilberry fruits collected in the woods of Kranj and Škofja Loka (Slovenia), was made at the Faculty of Biotechnology in Ljubljana (Slovenia) using the method previously published by Može and co-workers [13]. The bilberry fruits (*Vaccinium myrtillus* L.) were identified at the Department of Food Science and Technology, Biotechnical Faculty, Slovenia. Briefly, 600 g of previously frozen bilberry was homogenized in 2 L of ice-cold deoxygenated methanol. The extraction was performed at ambient temperature by continuous stirring on a magnetic stirrer (IKA REO Basic C, Königswinter, Germany) for 60 min and followed by vacuum filtration through the technical filter paper. The residue was re-extracted in 1 L of ice-cold deoxygenated methanol for 30 min. The extracts obtained were then stored at −20 °C until analysis. In the further procedure, before in vivo experiments, the methanol in the bilberry extract was evaporated under decreased pressure at 40 °C to eliminate methanol and make the concentrated extract suitable for use in laboratory animals. Gas chromatography was used to show that no methanol was present in the concentrated bilberry extract [14].

Individual anthocyanins in the bilberry extract were analyzed using LC-MS/MS with SPE, using the method according to Može and co-workers [13]. Briefly, after SPE, the LC-MS/MS analysis was performed on a system that consisted of an Agilent 1100 binary pump and autosampler (Phenomenex, Torrance, CA, USA) coupled to a Micromass Quattro Micromass spectrometer equipped with an electrospray ionizer source (Waters, Milford, MA, USA), using Gemini C18 column (150 × 2 mm, 3 μm) protected by Gemini C18 Security Guard cartridge (4 × 2 mm) (Phenomenex, Torrance, CA, USA). Each sample was analyzed in three replicates. The limit of detection (LOD), quantification (LOQ), and repeatability (CV) were determined based on the previously described method [13]. The quantification of individual anthocyanins was calculated according to external standard, cyanidin 3-glucoside, with the calibration curve ranging from 0.5 to 45 mg/L. The contents were expressed as μg of the standard cyanidin 3-glucoside equivalents per 1 L of the extract.

### 2.3. Animals and Housing

Male 10-week-old Wistar rats weighing 230–250 g, bred at the Research Center for Biomedicine (Faculty of Medicine, University of Nis, Nis, Serbia), were housed in plastic cages with ad libitum access to food and drinking water. The animals were kept in a controlled environment at 20 ± 2 °C, with 55% relative humidity, central ventilation, and a 12 h light/dark cycle. The experiment was carried out in compliance with the Guide for the Care and Use of Laboratory Animals published by the National Academy of Sciences and ARVO Statement for the Use of Animals in Ophthalmic and Vision Research. All experimental procedures with the laboratory animals were performed according to the National Institutes of Health guide (NIH), and permission was obtained from the Ethics Committee of the Faculty of Medicine in Niš (01-9337-16 and 01-10204-3).

### 2.4. Establishing Animal Model of Hyperglycemia

Rats were divided randomly into four (I to IV) equal groups of 7 rats each. To induce hyperglycemia, the rats in groups III and IV were injected intraperitoneally (i.p.) with STZ dissolved in 0.1 M citrate buffer, pH 4.5, at a single dose of 45 mg/kg, followed by i.p. injection of NA at dose 110 mg/kg in saline solution after overnight fasting, following some adaptations of the model already described [14]. Animals from groups I and II received a single i.p. injection of citrate buffer and saline solution.

The non-fasting and fasting blood samples were taken on the 3rd and 7th days after the animals received STZ/NA injections using the glucose monitor set Accu-check Performa (Roche Diagnostics, Indianapolis, IN, USA) to confirm hyperglycemia. The animals were considered hyperglycemic if the non-fasting and/or fasting blood glucose levels were >8.5 mmol/L and >7.1 mmol/L, respectively.

### 2.5. Bilberry Extract Treatment

The experimental groups were divided as follows: I—control (C); II—control + bilberry extract (C + BE); III—STZ/NA; and IV—STZ/NA + bilberry extract (STZ/NA + BE). The daily intake of anthocyanins was recommended to be 20 mg per animal, which corresponded to a dose of 50 mg/kg [15]. The concentration of anthocyanins in the drinking water was 0.45 mg/mL. The anthocyanin intake was influenced by the water intake, but not significantly since the water intake was closely monitored and did not differ significantly between bilberry-treated groups. The extract was administered orally in drinking water for 14 days to groups II and IV, 4 weeks after the induction of hyperglycemia, when the model was considered to be fully developed. Food and water consumption in each group of animals were measured daily. The body weight was measured weekly after overnight fasting.

### 2.6. Sample Collection and Preparation

At the end of the experimental period, non-fasting and fasting tail-vein blood samples were collected for blood sugar measurement using glucose meter Accu-check Performa (Roche Diagnostics, Indianapolis, IN, USA). After an overnight fast, the rats were anesthetized with ketamine (Ketamidor, Richter pharma ag; 100 mg/kg, i.p.), and blood for biochemical analysis was collected via cardiac puncture. Serum and plasma were separated by centrifugation for 15 min at 3000 rpm at room temperature.

Quickly after exsanguination, retinas were dissected [16], weighed, and then homogenized in an ice-cold distilled water using a mini homogenizer (Homogenisator Mixy Mini cordless grinder, Nippon genetics). Retinal homogenates were then centrifuged at 4000 rpm for 10 min at 4 °C, and the clear supernatants were stored at −80 °C for further biochemical analysis. The protein concentration in homogenate was determined by using the method described by Lowry [17], using a bovine serum albumin as the standard.

### 2.7. Determination of Serum Parameters

Serum glucose levels, total cholesterol (TC), HDL-cholesterol, and triglycerides were determined with enzymatic methods on an automated chemistry analyzer (Dimension RxL Max, Siemens, Deerfield, IL, USA), using original reagents from Siemens Healthcare Diagnostics. Insulin levels were determined by using a commercially available rat enzyme-linked immunosorbent assay (ELISA) kit (Mercodia, Upsala, Sweden).

### 2.8. Determination of Thiobarbiturate Reacting Substances

The level of thiobarbituric reacting substances (TBARSs) in the retina was determined by the spectrophotometric method by Andreeva and co-workers [18]. The color intensity of the chromogen formed in this reaction was measured at 532 nm. The TBARS concentration was calculated using a molar extinction coefficient equal to 1.54 × 10^5^ M^−1^cm^−1^, and the amount was expressed in mmol per mg of proteins.

### 2.9. Determination of Advanced Oxidative Protein Products

The level of advanced oxidative protein products (AOPPs) was determined by the spectrophotometric method previously described [19]. Calibration was carried out using chloramine-T solution, with the absorbance measurement at 340 nm in the presence of potassium iodide. The AOPP concentration is expressed in μmol/L chloramine-T equivalent and then converted to mg of protein.

### 2.10. Determination of iNOS, VEGF, and MMP-9 in Retinal Homogenates

The levels of iNOS were determined by using a commercially available rat enzyme-linked immunosorbent assay (ELISA) kit (Rat Inducible Nitric Oxide Synthase ELISA Kit, Cusabio College Park, MD, USA). The VEGF levels were determined by using a commercially available rat ELISA kit (Quantikine ELISA Rat VEGF, R&DSystems, Minneapolis, MA, USA). The MMP-9 levels were determined by using a commercially available rat ELISA kit (Quantikine ELISA Rat MMP-9, R&DSystems, Minneapolis, MA, USA).

### 2.11. Statistical Analysis

Data are expressed as the mean ± standard deviation. The Shapiro–Wilk test was used to test the normality of distribution. Comparisons of continuous variables were analyzed by using the one-way ANOVA, followed by Tukey’s post hoc test. Statistical significance (*p*) was set at *p* < 0.05.

## 3. Results

### 3.1. Anthocyanin Content in the Bilberry Extract

**Figure 1:**
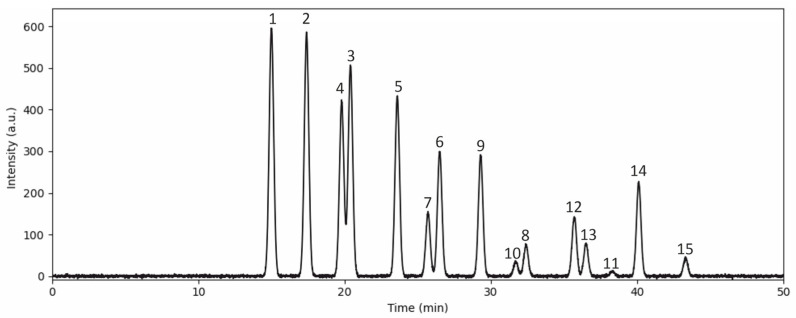
Representative chromatogram of bilberry extract with anthocyanins labeled numerically as given in Table 1.

The anthocyanin concentration was determined using LC-MS/MS (Figure 1) and quantified as mg of cyanidin 3-glucoside equivalents per L of bilberry extract or 100 g of fresh bilberries. The extract contained 3895.2 ± 53.7 mg/L of anthocyanin equivalents in particular glucose-, galactose-, and arabinose-bound delphinidin (43.3%); cyanidin (29.6%); petunidin (13.5%); malvidin (8.9%); and peonidin (4.9%) (Table 1).

### 3.2. Effect of Bilberry Extract on Body Weight, Food, and Water Intake

The body weights of control and experimental rats at the beginning (initial body weight) and the end of the bilberry extract treatment (final body weight) are given in Table 2. No significant differences in body weight were observed among groups during the course of the experiment.

On the other hand, food and water intake were found to be higher in the STZ/NA group when compared to the control (*p* < 0.001). Bilberry extract application significantly affected both food (*p* < 0.001) and water intake (*p* < 0.01) in rats with hyperglycemia when compared to the animals with hyperglycemia that did not receive any kind of treatment. However, there were no significant differences in food and water intake in group STZ/NA + BE compared to C and C + BE groups (Table 2).

### 3.3. Effect of Bilberry Extract on Serum Glucose and Insulin

**Figure 2:**
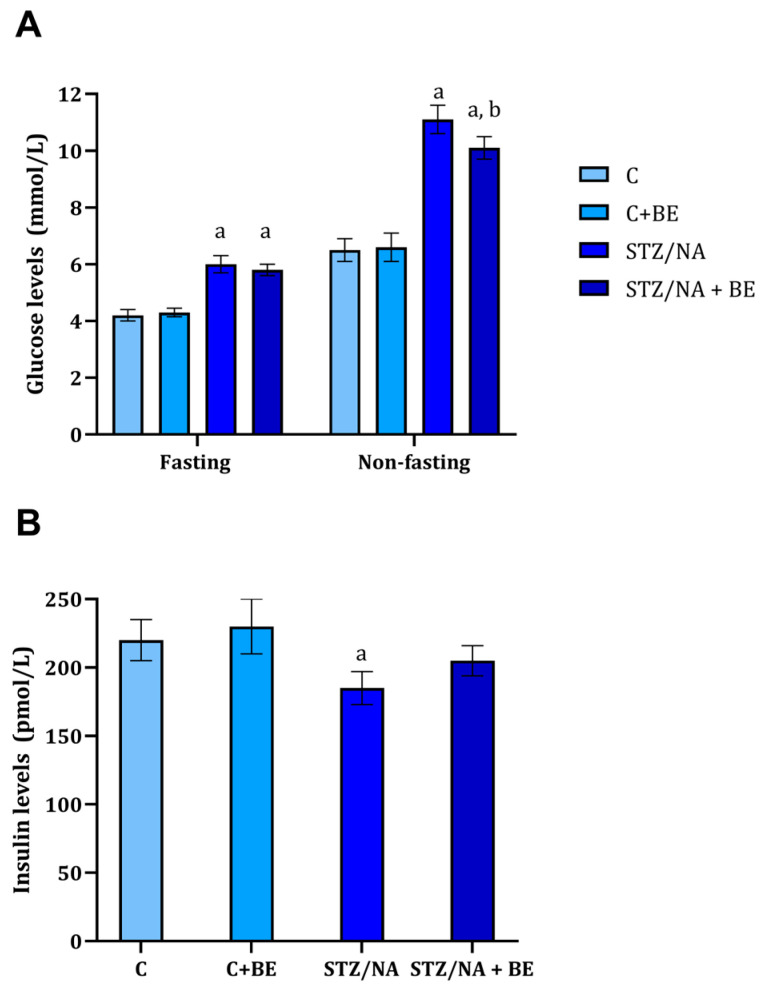
The concentration of fasting and non-fasting blood glucose (**A**) and insulin (**B**) in rats with STZ/NA-induced hyperglycemia treated with bilberry extract (BE) in the dose of 50 mg/kg of anthocyanin equivalents for 14 days at the end of the treatment period. Values are given as mean ± SD. The difference between groups was determined by one-way ANOVA, followed by Tuckey’s post hoc test; ^a^ *p* < 0.001 vs. control group (C); ^b^ *p* < 0.05 vs. STZ/NA group.

Non-fasting and fasting glucose levels in control and experimental rats are shown in Figure 2A. There were significant differences in both non-fasting and fasting glucose levels between the STZ/NA group and the control group. Non-fasting glucose levels in the STZ/NA group were significantly higher when compared to the STZ/NA + BE group (*p* < 0.05). However, bilberry extract had no effect on the fasting glucose when compared to the STZ/NA group (Figure 2A). Glucose levels were significantly higher compared to the control group (Figure 2A).

Fasting insulin levels are shown in Figure 2B. As expected, insulin was significantly lower in the STZ/NA group when compared to the control (STZ/NA vs. C, *p* < 0.05). Insulin level did not differ in bilberry extract-treated groups when compared to appropriate control both healthy and the one with experimentally induced diabetes (C + BE vs. C, NS; STZ/NA + BE vs. STZ/NA, NS).

### 3.4. Effect of Bilberry Extract on Lipid Profile

**Figure 3:**
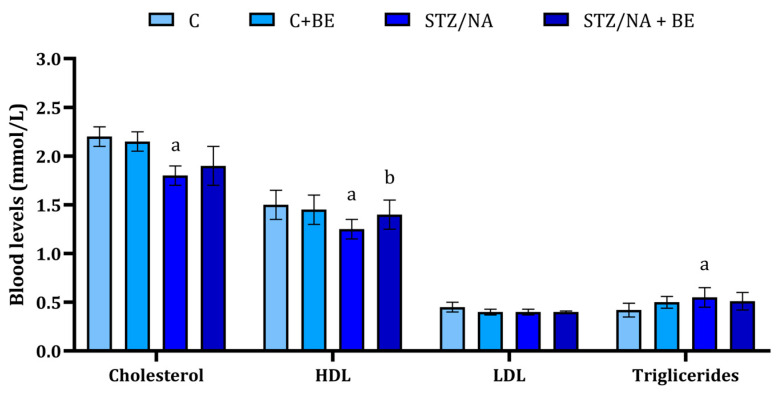
The concentration of serum lipids the in rats with STZ/NA-induced hyperglycemia treated with bilberry extract (BE) in the dose of 50 mg/kg of anthocyanin equivalents for 14 days at the end of the treatment period. Values are mean ± SD. The difference between groups was determined by one-way ANOVA, followed by Tuckey’s post hoc test; ^a^ *p* < 0.05 vs. control; ^b^ *p* < 0.05 vs. STZ/NA group.

Total cholesterol in the STZ/NA group was found to be lower when compared to the control group (Figure 3); at the same time, bilberry extract prevented the change in total cholesterol levels in animals with hyperglycemia (STZ/NA + BE group). The levels of HDL were also found to be significantly lower in STZ/NA group when compared to the control. The application of bilberry extract also significantly reduced HDL compared to the STZ/NA group (Figure 3). The levels of LDL did not vary between the control and STZ/NA groups; however, they were lower in the STZ/NA + BE group when compared to the STZ/NA group (*p* < 0.05, Figure 3). Finaly, triglyceride levels did not differ significantly between the examined groups (Figure 3).

### 3.5. Effect of Bilberry Extract on TBARS Levels in Retinal Tissue

**Figure 4:**
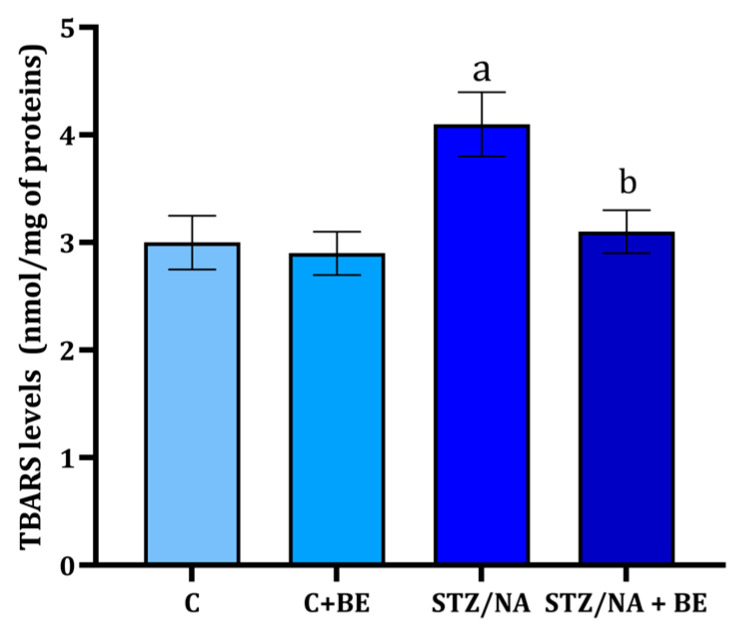
The concentration of thiobarbituric acid reactive substances (TBARSs) in the retina of rats with STZ/NA-induced hyperglycemia treated with bilberry extract (BE) in the dose of 50 mg/kg of anthocyanin equivalents for 14 days at the end of the treatment period. Values are given as mean ± SD. The difference between groups was determined by one-way ANOVA, followed by Tuckey’s post hoc test; ^a^ *p* < 0.05 vs. control group (C); ^b^ *p* < 0.05 vs. STZ/NA group.

In the control group and the group receiving BE levels of TBARS, around 3 nmol/mg of tissue proteins was present (Figure 4). TBARS levels in the retina were found to be significantly higher in the STZ/NA group when compared to the control group (Figure 4). The levels of TBARS were significantly lower in STZ/NA + BE when compared to STZ/NA animals, and at the same time, they were not different from the ones in the control group (Figure 4).

### 3.6. Effect of Bilberry Extract on AOPP in the Retinal Tissue

**Figure 5:**
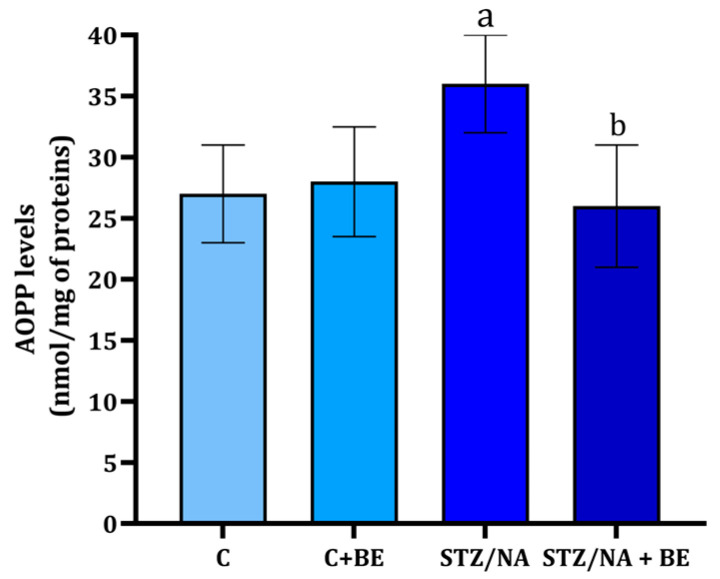
The concentration of advanced oxidized protein products (AOPPs) in the retina of rats with STZ/NA-induced hyperglycemia treated with bilberry extract (BE) in the dose of 50 mg/kg of anthocyanin equivalents for 14 days at the end of the treatment period. Values are given as mean ± SD. The difference between groups was determined by one-way ANOVA, followed by Tuckey’s post hoc test; ^a^ *p* < 0.001 vs. control group (C); ^b^ *p* < 0.001 vs. STZ/NA group.

The AOPP levels in the retina were found to be higher in the STZ/NA group when compared to the control group (Figure 5; *p* < 0.001). The AOPP levels were at the same time statistically significantly lower in STZ/NA + BE animals when compared to STZ/NA animals that received no treatment (*p* < 0.001). Application of BE to rats with STZ/NA (STZ/NA + BE) completely prevented the change in AOPP retina levels (Figure 5).

### 3.7. Effect of Bilberry Extract on iNOS in the Retinal Tissue

The activity of iNOS in the retina was found to be higher in the STZ/NA group when compared to the control group (Table 3, *p* < 0.05). At the same time, iNOS activity did not differ in STZ/NA + BE animals when compared to STZ/NA animals that received no treatment (Table 3).

### 3.8. Effect of Bilberry Extract on VEGF in the Retinal Tissue

The levels of VEGF in the retinal tissue were found to be significantly higher in the STZ/NA group when compared to the control group (Table 3). At the same time, VEGF levels were significantly lower in STZ/NA + BE animals when compared to the group of STZ/NA animals (Table 3). The values of VEGF were almost at the level of those seen in the control and BE-treated group.

### 3.9. Effect of Bilberry Extract on MMP-9 in the Retinal Tissue

The MMP-9 amounts in the control group and group treated with BE were between 47 and 60 pg/mg of protein tissue (Table 3). The MMP-9 levels in the retina were significantly higher in the STZ/NA than in the control group (Table 3). The MMP-9 levels were significantly lower in STZ/NA + BE animals when compared to STZ/NA animals that received no treatment (Table 3, *p* < 0.01).

## 4. Discussion

The most important risk factors for progression in vision loss associated with diabetic retinopathy include the duration of diabetes and hyperglycemia, and the presence of hypertension [20]. Hyperlipidemia has been mentioned to be one of the additional risk factors for the clinical progression of diabetic retinopathy [3]. Our study assessed the effects of bilberry extract treatment on serum glucose and lipids, identified modifiable risk factors for the progression of diabetic retinopathy. Hypoglycemic effects of the anthocyanin-rich extract were previously demonstrated in humans [21] and animals [10,22], and the obtained results suggest the hypoglycemic effect of bilberry extract on non-fasting blood glucose. However, these prior studies did not demonstrate significant effects on fasting blood glucose. The reason behind these findings might be associated with the applied daily dose. In the present study, the approximate dose of 50 mg/kg of anthocyanins was used, whereas the study that demonstrated the effect of bilberry on fasting glucose and insulin fed the animals bilberry powder (2 g/day) containing 45 mg of anthocyanins/500 g of powder [10]. The obtained results regarding glycemia were further corroborated in the study where the bilberry extract (100 mg/k g of anthocyanins) affected fasting glycemia [8]. The hypoglycemic effect of bilberry might be achieved by affecting insulin secretion and possibly glucose transport. Anthocyanins were found to stimulate insulin secretion in vitro, with cyanidins and delphinidins showing the most prominent effect [23]. Interestingly, although the STZ/NA-induced statistically significant changes in insulin levels, the levels were objectively not much lower than in the control/experimental group, which might be the consequence of the lower dose of STZ applied. Also, low-bush blueberry extract was demonstrated to enhance glucose transport into muscle cells and adipocytes in the absence of insulin [24]. Although the results did not demonstrate the significant effect of bilberry extract application on serum insulin levels (Figure 2B), significant effects on non-fasting hyperglycemia, which might be, at least partially, mediated by an increase in glucose uptake, were found.

It was previously reported that bilberry extract applications in STZ-treated diabetic rats produce hyperlipidemic effects [10]. These effects were observed through the reduction in total cholesterol, LDL-C, VLDL-C, and triglyceride levels, together with the prevention of HDL-C decline [10]. The present study demonstrated the effect of bilberry extract on dyslipidemia, characterized by the significant decrease in HDL level, which was reversed with bilberry extract treatment. Also, the bilberry extract application caused a decrease in triglycerides. In diabetes, there is evident alteration in lipid metabolism which reflects insulin resistance and is potentially associated with uncontrolled fat deposit degradation, all a consequence of increased energy demands that follow glucose metabolism impairment. The ability of bilberry extract to normalize insulin release and normalize the glucose blood levels in rats treated with STZ/NA might prevent lipid metabolism alterations.

The results of the present study demonstrated the potential for preventing oxidative retinal-tissue damage of bilberry extract in rats with hyperglycemia. Antioxidant properties of bilberry extract have already been documented in vitro [11], and in some cases, they have been associated with its in vivo effects [25]. Bilberry extract application prevented an increase in retinal-tissue TBARS and AOPP in hyperglycemic animals. Similar results regarding the effect of bilberry extract on oxidative stress (MDA) in the retina were reported in the visible-light-induced retinal degeneration model in pigmented rabbits [26]. The ability of bilberry extract to prevent TBARS and AOPP generation can be partially connected to the ability of anthocyanins to scavenge ROS [11,12].

It has been postulated that NO signaling plays a role in diabetic retinopathy, during which hyperproduction of NO, by either form of NOS, damages retinal cells (decreased viability, increases leukocyte migration, and alters blood vessel permeability [27]. In a mouse model of uveitis induced by lipopolysaccharide, the application of bilberry extract mitigated retinal NO signaling and thus contributed to the general inflammatory process affecting eyes [15]. Also, in other models, such as cardiac damage, bilberry extract was proved to be effective in decreasing/altering NO signaling [28]. The question remains, are the effects of bilberry extract on NO signaling tissue-specific or dose-related? This can be observed in light of the effects of bilberry non-fasting glycemia, which might be absent due to low applied dose of bilberry extract.

Apart from its potential to cause damage to cells’ structural molecules, the generated ROS is a strong stimulus for the release of pro-inflammatory cytokines and angiogenic parameters, such as VEGF, which further play an important role in the pathogenesis of diabetic retinopathy [4,26]. Some of the main events associated with the progression of diabetic retinopathy are blood–retinal barrier destruction and leakage, leading to enhanced angiogenesis. In this process, both VEGF and MMPs are known to play an important role [29]. VEGF is a potent and vital factor influencing angiogenesis and vascular permeability. Its elevated retinal-tissue levels correspond with blood–retinal barrier breakdown in both animals and humans, with a detected positive correlation between VEGF levels and the vascular permeability in the retina [8]. The administration of bilberry extract for 14 days was found to significantly reduce the level of retinal VEGF and MMP-9 (Table 3). A similar effect of bilberry extract on retinal VEGF was previously reported in the visible-light-induced retinal degeneration model [26], and its lower expression in the retina of diabetic rats treated with bilberry extract was also reported [8].

On the other hand, MMPs, secreted by endothelial cells, are hypothesized to play a key role in the processes of extracellular matrix remodeling during angiogenesis and disruption of the blood–retinal barrier [6]. This enzyme is also involved in the degradation of the extracellular matrix and can cleave cytokine precursors such as membrane-bound VEGF into active forms, leading to the increased bioavailability for its receptor, thus potentiating the VEGF effect on blood–retinal barrier disruption and angiogenesis [30]. The impact of bilberry extract on MMP-9 has been previously shown in an in vitro study, suggesting that it may inhibit its activity [31]. Through this mechanism, bilberry could partially prevent retinal-tissue remodeling and complications related to vascular proliferation.

## 5. Conclusions

In summary, bilberry extract exerted beneficial effects on the retinal-tissue biochemical changes in rats with STZ/NA hyperglycemia by affecting risk factors for the development of diabetic retinopathy, i.e., hyperglycemia and lipid levels. Furthermore, bilberry extract treatment ameliorated oxidative damage in the retina (measured through TBARS and AOPP levels) and reduced VEGF and MMP-9 levels, positively affecting blood–retinal barrier integrity and angiogenesis, potentially preventing or delaying the progression of diabetic retinopathy. Therefore, the application of bilberry extracts might be considered in the prevention and early treatment of diabetic retinopathy.

## Figures and Tables

**Table 1:** The anthocyanin composition and content in the bilberry extract used in this experiment.

No.	Anthocyanins	mg/L	mg/100 g	%	%
**1**	**Delphinidin 3-galactoside**	595.98 ± 0.9	132.05 ± 0.2	15.3	43.3
**2**	**Delphinidin 3-glucoside**	584.29 ± 1.0	129.46 ± 0.2	15.0	
**3**	**Delphinidin 3-arabinoside**	506.38 ± 1.3	112.20 ± 0.4	13.0	
**4**	**Cyanidin 3-galactoside**	420.69 ± 1.2	93.21 ± 0.3	10.8	29.6
**5**	**Cyanidin 3-glucoside**	432.37 ± 0.5	95.80 ± 0.20	11.1	
**6**	**Cyanidin 3-arabinoside**	299.93 ± 0.3	66.45 ± 0.1	7.7	
**7**	**Petunidin 3-galactoside**	151.91 ± 0.8	33.66 ± 0.2	3.9	13.5
**8**	**Petunidin 3-arabinoside**	74.01 ± 0.3	16.39 ± 0.1	1.9	
**9**	**Petunidin 3-glucoside**	292.14 ± 6.4	64.73 ± 1.4	7.5	
**10**	**Peonidin 3-galactoside**	35.05 ± 0.2	7.76 ± 0.1	0.9	4.9
**11**	**Peonidin 3-arabinoside**	11.68 ± 0.2	2.58 ± 0.1	0.3	
**12**	**Peonidin 3-glucoside**	144.12 ± 0.3	31.93 ± 0.1	3.7	
**13**	**Malvidin 3-galactoside**	77.90 ± 0.2	17.26 ± 0.3	2.0	8.9
**14**	**Malvidin 3-glucoside**	225.92 ± 0.8	50.05 ± 0.1	5.8	
**15**	**Malvidin 3-arabinoside**	42.84 ± 1.2	9.49 ± 0.3	1.1	
	**Total**	3895.2 ± 53.7	863.4 ± 11.5	100	100

Values are mean ± SD.

**Table 2:** Average initial and final body weight, food and water consumption per animal in control, and STZ/NA rats treated with bilberry extract (BE) in the dose of 50 mg/kg of anthocyanin equivalents for 14 days in the beginning and at the end of the treatment period.

Parameters/Group	I (C)	II (C + BE)	III (STZ/NA)	IV (STZ/NA + BE)
**Initial body weight (g)**	351 ± 7	365 ± 15	352 ± 14	351 ± 10
**Final body weight (g)**	395 ± 12	400 ± 12	413 ± 19	401 ± 21
**Food consumption (g/day)**	30 ± 2	31 ± 2	36 ± 3 ^a^	30 ± 4 ^b^
**Water consumption (mL/day)**	41 ± 4	41 ± 6	56 ± 6 ^a^	45 ± 7 ^b^

Values are presented as mean ± SD. The difference between groups was determined by one-way ANOVA, followed by Tuckey’s post hoc test; ^a^ *p* < 0.05 vs. control; ^b^ *p* < 0.05 vs. STZ/NA group.

**Table 3:** The activity of inducible nitric oxide synthase (iNOS) and concentration of vascular endothelial growth factor (VEGF) and matrix metalloproteinase 9 (MMP-9) in the retina of control and STZ/NA rats treated with bilberry extract (BE) in the dose of 50 mg/kg of anthocyanin equivalents for 14 days, at the end of the treatment period.

Parameter/Group	I (C)	II (C + BE)	III (STZ/NA)	IV (STZ/NA + BE)
**iNOS (IU/mg)**	13.9 ± 0.9	14.1 ± 1	17.2 ± 0.9 ^a^	14.9 ± 0.8
**VEGF (pg/mg)**	22.2 ± 3.6	20.9 ± 1.9	27.5 ± 2.5 ^a^	23.9 ± 1.7 ^b^
**MMP-9 (pg/mg)**	47.4 ± 15.1	60.3 ± 7.6	86.4 ± 8.6 ^a^	41.2 ± 8.2 ^b^

Values are presented as mean ± SD. The difference between groups was determined by one-way ANOVA, followed by Tuckey’s post hoc test; ^a^ *p* < 0.05 vs. control; ^b^ *p* < 0.05 vs. STZ/NA group.

## Data Availability

Data will be made available upon reasonable request to the corresponding author.
